# Contributions of Gray Matter Microstructure to Differences in Fluid Cognition and Episodic Memory Across the Healthy Adult Lifespan

**DOI:** 10.1002/hbm.70511

**Published:** 2026-03-23

**Authors:** Jenna L. Merenstein, Ilana J. Bennett, David J. Madden

**Affiliations:** ^1^ Department of Psychology University of Utah Salt Lake City Utah USA; ^2^ Department of Psychology University of California Riverside California USA; ^3^ Brain Imaging and Analysis Center Duke University Medical Center Durham North Carolina USA; ^4^ Department of Psychiatry and Behavioral Sciences Duke University Medical Center Durham North Carolina USA

**Keywords:** adult lifespan, cognitive performance, diffusion‐weighted imaging (DWI), moderation analyses, neurite orientation density and dispersion index (NODDI)

## Abstract

Cognitive decline, in healthy older adults without cognitive impairment or dementia, has been associated with numerous microstructural alterations in brain tissue using magnetic resonance imaging (MRI). Prior studies have primarily linked age‐related cognitive decline to alterations in white matter tissue, but methodological advances in diffusion‐weighted imaging (dMRI) data acquisition and modeling now allow for these analyses to be extended to gray matter tissue. Here, using a sample of 152 healthy adults (18–88 years of age), we used a multicompartment dMRI model to assess (1) age‐related differences in gray matter microstructure of functionally defined networks and (2) whether microstructural alterations accounted for age‐related differences in episodic memory and speed‐dependent fluid cognition. We observed significant age‐related alterations in gray matter tissue in the form of nonlinear, age‐related increases and decreases in intracellular and dispersed diffusion, respectively, and linear increases in free diffusion. Free diffusion exhibited the most pronounced age‐related effects, especially for frontoparietal relative to occipital regions. Dispersed diffusion in the dorsal attention network statistically mediated age‐related differences in episodic memory performance. Moreover, higher intracellular diffusion in the default mode and ventral attention networks was related to worse fluid cognition performance, but only for adults > 51 years of age. These results suggest that healthy aging is accompanied by distinct profiles of gray matter microstructural alterations that negatively affect memory and speed‐dependent cognition, the latter of which is more pronounced after midlife.

## Graphical abstract

Multicompartment measures of gray matter microstructure and fluid cognition and episodic memory performance were assessed across the adult lifespan. Age‐related effects were large and linear for free diffusion, moderate and nonlinear for intracellular diffusion, and weaker for dispersed diffusion, and these alterations helped explain age‐related differences in cognition.

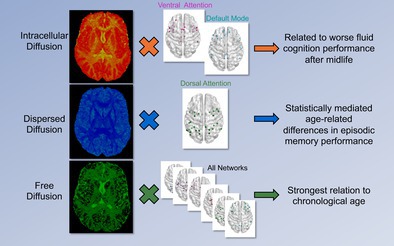

## 1 Introduction

Across the adult lifespan, healthy cognitive aging is accompanied by numerous microstructural alterations in the composition of brain tissue. Magnetic resonance imaging (MRI) studies, capitalizing on diffusion‐weighted imaging (DWI), have primarily investigated age‐related differences in cognitive abilities with microstructural alterations in white matter (WM) tissue (e.g., Agah et al. 2025; Madden et al. 2009; Pang et al. 2025; Peter et al. 2025), including properties such as fiber incoherence, decreased axonal diameter, and demyelination (Song et al. 2003; Wei et al. 2013). These findings support the cortical disconnection theory of aging, which proposes that WM degradation contributes to healthy aging (Bartzokis et al. 2004; Bennett and Madden 2014; Madden et al. 2017; O'Sullivan et al. 2001). Older adult age has also been associated with microstructural alterations in gray matter (GM) tissue, though to a lesser extent (e.g., Bouhrara et al. 2023; Greenman and Bennett 2025; Lee et al. 2024; Nazeri et al. 2015; Reas et al. 2020; Singh et al. 2025, 2024), including properties such as decreased dendritic complexity, neuronal shrinkage, and synaptic loss (Crombe et al. 2018). However, only a handful of studies have associated age‐related alterations in GM microstructure to cognitive performance in healthy adults. Better understanding of how in vivo proxies of GM microstructure differ across the adult lifespan and contribute to cognitive decline in healthy aging are necessary for informing disconnection theories of aging.

Studying age‐related alterations in the complex cytoarchitecture of GM using DWI has historically been difficult because extant (primarily tensor‐based) models were instead designed to study the consistent, highly aligned organization of WM fiber bundles (Beaulieu 2002; Mori and Zhang 2006). However, more accurate depictions of GM tissue can be obtained in vivo using multicompartment modeling techniques, such as the neurite orientation density and dispersion index (NODDI; Zhang et al. 2012), which estimate three sources of water diffusion within each voxel. This includes the intracellular volume fraction (FICVF) modeled as a set of sticks (labeled as the intracellular diffusion metric here), the orientation dispersion index (ODI) modeled as the dispersion of sticks (labeled as the dispersed diffusion metric here), and the fraction of isotropic diffusion (FISO) modeled as an isotropic sphere (labeled as the free diffusion metric here).

One recent longitudinal study using NODDI to study GM microstructure of the hippocampus in healthy aging reported increases in the dispersed diffusion metric and decreases in the intracellular diffusion metric in healthy older adults (Zia et al. 2025). Cross‐sectional studies have similarly identified age‐related alterations in hippocampal microstructure, which have been associated with episodic memory performance (Ibrahim and Bennett 2023; Radhakrishnan et al. 2022, 2020; Venkatesh et al. 2020). Age group differences in GM microstructure have also been reported within the basal ganglia and linked to age‐related differences in implicit associative learning (Franco et al. 2021). Beyond these deep GM structures, age‐related differences in cortical microstructure have been observed across the older adult lifespan (56–99 years of age; Reas et al. 2020) and the microstructure of dorsolateral prefrontal and cingulate regions helped explain memory performance within healthy older adults, as well as patients with mild cognitive impairment (Gozdas et al. 2021). Together, prior NODDI studies focused on older adults or comparing older adults to younger adults suggest that multicompartment diffusion modeling helps characterize age‐related differences in GM microstructure, especially in the hippocampus, and helps explain differences in memory performance.

Using an extension of the NODDI model that requires high‐gradient data acquisition (soma and neurite density imaging; Palombo et al. 2020), age‐related differences in soma‐specific sources of diffusion have been observed across the adult lifespan, although this study did not assess cognition (Lee et al. 2024). One subsequent study linked these soma‐specific sources of diffusion to age‐related differences in fluid cognition, but their analyses were restricted to motor and temporal GM regions (Singh et al. 2025). To our knowledge, only two studies have used the standard NODDI model to examine age‐related alterations in brain‐wide microstructure across the adult lifespan (Filip et al. 2023; Nazeri et al. 2015), and only one examined whether these alterations helped explain age‐related differences in cognitive measures of executive function (Nazeri et al. 2015). Both studies also excluded the free diffusion metric, despite this metric exhibiting large differences in healthy aging (Greenman and Bennett 2025). Because Nazeri et al. (2015) did not assess memory performance, it remains unclear whether age‐related alterations in GM microstructure have greater impacts on certain cognitive domains. Finally, the Nazeri et al. (2015) study (and those reviewed above) assessed age‐related differences in GM microstructure using the default NODDI parameter for intrinsic diffusivity, but subsequent work has shown that this parameter is better optimized for WM than for GM (Fukutomi et al. 2019, 2018; Guerrero et al. 2019). One recent NODDI study assessed age group differences in GM microstructure using an intrinsic diffusivity parameter optimized for GM (Greenman and Bennett 2025), but these authors did not include middle‐aged adults (i.e., data were not collected for adults 29–64 years of age) or assess whether these optimized estimates of GM microstructure help explain age‐related cognitive decline.

Here, we acquired multicompartment DWI data from 152 participants across the adult lifespan (18–88 years of age) who completed an extensive cognitive task battery assessing general speed‐dependent fluid cognition and episodic memory. Using an intrinsic diffusivity parameter optimized for GM tissue, we aimed to replicate (1) larger age‐related differences for free diffusion relative to the intracellular or dispersed diffusion metric and (2) stronger age‐related effects on frontoparietal relative to occipital cortical regions (Greenman and Bennett 2025). We extend previous work by including adults across the entire adult lifespan, which allowed us to conduct a novel examination of the relative linearity of age‐related differences in these more optimized NODDI measures of GM microstructure. In addition, we assessed both general fluid cognition and episodic memory in the same study. Based on prior work (Gozdas et al. 2021; Nazeri et al. 2015), we expected that age‐related differences in cortical microstructure, especially the dispersed diffusion metric, would account for age‐related variance in both domains of cognition. Finally, we tested the novel hypothesis that the relation between GM microstructure and cognitive performance may differ as a function of chronological age, with a larger effect observed for middle‐age and older adults than for younger adults. This study represents the first brain‐wide examination of three optimized NODDI measures of GM microstructure in a well‐characterized sample of adults across the lifespan and how these measures relate to fluid cognition and episodic memory. By using a biophysically informed model, results will provide additional anatomical specificity to findings from previous studies using model‐free mathematical frameworks to assess GM microstructure (Bouhrara et al. 2023) and cognition (Singh et al. 2024) in healthy aging.

## 2 Materials and Methods

### 2.1 Participants

This study was conducted in compliance with the Code of Ethics of the World Medical Association (Declaration of Helsinki) for experiments involving humans and was approved by the Institutional Review Board for Duke University Medical Center. All participants provided informed consent and received monetary compensation for their participation.

Participants completed an initial, neurocognitive testing session (approximately 2 h), which included both psychometric screening tests and a battery of 12 laboratory‐based tests of fluid cognition (as described in Section 2.2). This was followed by a 1.5 h. MRI scan approximately 1 month later (median interval = 28 days, range = 5–169 days). Two hundred and fifty‐two healthy adults between 18 and 88 years of age, who resided in the Durham–Chapel Hill–Raleigh area, completed the initial neurocognitive testing session. Recruitment efforts included extant participant pools and registries, electronic social media advertisements, and physical flyers posted around the campus of Duke University Medical Center. Individuals included in the final sample reported that they had completed at least a high school education and were free of major neurological (e.g., epilepsy, stroke) and other medical (e.g., diabetes, emphysema, uncontrolled hypertension) conditions. All individuals (including younger, middle‐age, and older adults) were screened for general cognitive performance using either the Mini‐Mental State Exam (MMSE; score ≥ 27; *n* = 68; Folstein et al. 1975) or the Montreal Cognitive Assessment (MoCA; score ≥ 26; *n* = 84; Nasreddine et al. 2005). The current study combined data from two separate experiments, where the primary change in protocol was that participants in the first study (*n* = 68) completed the MMSE and participants in the second study (*n* = 84) instead completed the MoCA due to its increased sensitivity to general cognition (Freitas et al. 2013). These tests were used here solely for screening purposes and were only analyzed for their relation to age, separately within each subsample. Individuals of all ages were further screened for general intelligence using the vocabulary subtest of the Wechsler Adult Intelligence Scale (WAIS)‐III (score > 50th percentile; Wechsler 1997), depression using the Beck Depression Inventory (score ≤ 16; Beck 1978), and visual acuity using the Freiburg Visual Acuity Test (Snellen score < 20/42; Bach 1996). Sixty‐three individuals were excluded for failing to meet the initial screening criteria or MRI safety requirements (e.g., claustrophobia, pacemaker/stent, or ferrous metal implant), and eight were lost to attrition. Two additional individuals were excluded for being outliers (> 3 standard deviations) on one or more of the cognitive tests (described below, Section 2.2).

The remaining 179 participants who passed the screening also completed MRI scanning. However, 27 participants were excluded because of excessive head motion, image artifacts or processing issues, or poor performance (accuracy < 75%) on a visual search task completed during task‐related fMRI scanning. Although the visual search task is not included in the present report, we continued to exclude these participants to remain consistent with our previously published work on subsets of this sample (Madden et al. 2024; Merenstein, Mullin, et al. 2023; Merenstein, Song, et al. 2025; Merenstein, Zhao, et al. 2025; Merenstein, Zhao, et al. 2023). Details on the final sample of 152 participants 18–88 years of age are presented in Table 1 (53.3% female, 7.9% Hispanic, 84% White). The age distribution of participants is as follows: 18–29 (*n* = 26), 30s (*n* = 25), 40s (*n* = 23), 50s (*n* = 20), 60s (*n* = 22), 70s (*n* = 22), and 80s (*n* = 14).

**TABLE 1:** Participant characteristics.

Measure	Mean	(SD)	*r* with age	*p*
Education (years)	17.16	(2.23)	0.06	0.480
MMSE	29.59	(0.60)	0.00	0.960
MoCA	**28.26**	**(1.23)**	**−0.34**	**< 0.001**
BDI	3.51	(3.74)	0.02	0.849
Vocabulary	56.16	(4.73)	−0.02	0.838
Color vision	**13.92**	**(0.36)**	**−0.23**	**< 0.001**
Visual acuity	**−0.01**	**(0.10)**	**−0.37**	**< 0.001**
ADI	**34.64**	**18.83**	**−0.30**	**0.005**

*Note: n* = 152 for all tests except the national percentile of area deprivation index based on current residential postcodes (*n* = 80; ADI; Kind and Buckingham 2018), Mini‐Mental State Exam (*n* = 68; MMSE; Folstein et al. 1975), and Montreal Cognitive Assessment (*n* = 84; MoCA; Nasreddine et al. 2005). Values are presented as mean (standard deviation, SD). BDI = score on the Beck Depression Inventory (Beck 1978, 2087; Beck 1978); Vocabulary = raw score on the vocabulary subtest of the Wechsler Adult Intelligence Scale III (Wechsler 1997); Color Vision = score on Dvorine (1963) color plates; Visual Acuity = inversed logarithm of the minimum angle of resolution for the Freiburg Visual Acuity Test (Bach 1996). The MMSE was completed by 28 younger adults (18–39 years of age), 24 middle‐age adults (40–64 years of age), and 16 older adults (65+ years of age). The MoCA was completed by 23 younger adults, 32 middle‐age adults, and 29 older adults. Significant correlations with age (uncorrected *p* < 0.05) are presented in bold.

### 2.2 Cognitive Testing

The present battery of cognitive tasks has been described in previous studies using a subset of this sample (Madden et al. 2024; Merenstein, Mullin, et al. 2023; Merenstein, Zhao, et al. 2025; Merenstein, Zhao, et al. 2023). Performance was assessed using computerized reaction time (RT), standardized psychometric measures, or measures from the National Institutes of Health (NIH) Toolbox (Gershon et al. 2013). Briefly, the battery comprised the following 12 individual tests: California Verbal Learning Task (~20‐min delayed recall measure), WAIS logical memory (~20‐min delayed recall measure), a nonverbal working memory change detection task (hits minus misses), NIH Toolbox Picture Sequence (theta score), simple and choice RT (median scores), neutral Flanker task trials (median RT divided by accuracy), Flanker score (incompatible minus compatible median RT), digit symbol substitution (median RT divided by accuracy), NIH Toolbox Pattern Comparison (number answered correctly in 85 s), Trail Making Test (time in seconds to complete part B vs. A), and the NIH Toolbox Dimensional Change Card Sort (computed score based on RT and accuracy).

### 2.3 MRI Data Acquisition

All imaging data were acquired at the Brain Imaging and Analysis Center at Duke University Medical Center from 2018 to 2024. Data for most participants (*n* = 100) were acquired on a standard 3T GE MR750 whole‐body MRI scanner equipped with an eight‐channel head coil. After performing scanner upgrades in 2022, the remaining data (*n* = 52) were acquired on a 3T GE Ultra High‐Performance MRI scanner equipped with a 48‐channel head coil. Participants wore earplugs to reduce scanner noise, and foam pads were used to minimize head motion.

For 68 participants, a high‐resolution T1‐weighted image volume was acquired using a 3D fast inverse‐recovery‐prepared spoiled gradient recalled (SPGR) sequence with the following parameters: echo time (TE) = 3.1 ms, repetition time (TR) = 2200 ms, flip angle = 8°, acquisition matrix = 512 mm^2^, field of view (FOV) = 240 mm^2^, voxel size = 0.5 mm^3^, number of slices = 292, and a sensitivity encoding (SENSE) factor of 2. For the remaining 84 participants, the parameters for the SPGR sequence were as follows: TR = 2132.8 ms, flip angle = 8°, acquisition matrix = 256 mm^2^, FOV = 256 mm^2^, voxel size = 1 mm^3^, and a SENSE factor of 2, with TE = 3.2 ms and number of slices = 116 (*n* = 9), TE = 3.2 ms and number of slices = 124 (*n* = 23), or TE = 3.0 ms and number of slices = 124 (*n* = 52).

For all 152 participants, whole‐brain DWI data were acquired using a single‐shot spin‐echo planar imaging (EPI) sequence with the following parameters: TE = 64.1 ms, TR = 4620 ms, flip angle = 90°, acquisition matrix = 144 mm^2^, FOV = 220 mm^2^, voxel size = 1.5 mm^3^, number of slices = 77, a SENSE factor of 1, and a multiband factor of 3. Diffusion‐weighted gradients were applied in 90 directions with *b* values of 1500 and 3000 s/mm^2^ and with two nondiffusion‐weighted *b* = 0 images. For most participants (*n* = 143), a second diffusion sequence was also acquired with six phase‐encoding directions of opposite polarity using identical parameters as the main DWI acquisition, except TR = 4971 ms. These scans of opposite polarity were used for the correction of susceptibility‐induced distortions in subsequent processing steps.

We also acquired data from the following imaging modalities: high‐resolution DWI, susceptibility weighted angiography, fluid attenuated inversion recovery, resting‐state functional MRI, and task‐related functional MRI while participants completed a visual attention task. Findings from these modalities have been, or will be, reported in separate articles (Madden et al. 2024; Merenstein, Mullin, et al. 2023; Merenstein, Zhao, et al. 2025; Merenstein, Zhao, et al. 2023).

### 2.4 Diffusion‐Weighted Imaging Data Processing and Modeling

#### 2.4.1 Preprocessing

All DWI data were preprocessed using MRtrix3 (Tournier et al. 2019) based on the following sequence of steps: denoising (*dwidenoise*); correction for motion, eddy current‐induced distortions, and susceptibility‐induced distortions (*dwifslpreproc;* which incorporates FSL's *topup*); bias‐correction (*dwibiascorrect*); and generation of a skull‐stripped whole‐brain mask (*dwi2mask*). All processed DWI scans were subjected to visual quality control and found to be acceptable for the degree of brain mask coverage, quality of motion and susceptibility‐induced distortion corrections, the presence of MR artifacts (e.g., ghosting, radio frequency inhomogeneities), and anatomical abnormalities.

#### 2.4.2 Model Fitting

We used the NODDI MATLAB toolbox (https://www.nitrc.org/projects/noddi_toolbox) to obtain diffusion estimates of GM microstructure (Zhang et al. 2012). This toolbox estimates a intracellular diffusion metric (i.e., FICVF or neurite density index [NDI]), a dispersed diffusion metric (i.e., ODI), and a free diffusion metric (i.e., FISO), modeled as a set of sticks, dispersion of the sticks, or an isotropic sphere, respectively. Based on our interest in GM rather than WM microstructure, the intrinsic diffusivity parameter was modified to 1.1 × 10^−3^ mm^2^/s to more accurately model the tissue compartments in GM (Crombe et al. 2018; Fukutomi et al. 2019, 2018; Greenman and Bennett 2025; Guerrero et al. 2019).

#### 2.4.3 Regions‐of‐Interest (ROIs)

We obtained NODDI diffusion estimates of microstructure from eight anatomical ROI masks. These were based on standard GM networks defined from resting‐state functional MRI data in a large independent sample (Yeo et al. 2011). The masks created here were comprised of nodes from the Brainnetome atlas (Fan et al. 2016) in Montreal Neurological Institute (MNI) 152 template space (1 mm^3^ resolution), including 210 cortical and 36 subcortical nodes. Each cortical node was combined into separate binary masks depending on its inclusion in one of the following seven cortical networks: dorsal attention, default mode, frontoparietal, limbic, sensorimotor, ventral attention, and visual networks (Yeo et al. 2011). The 36 subcortical nodes were combined into one subcortical network mask, similar to prior work (Capogna et al. 2022; Luo et al. 2020; Merenstein, Zhao, et al. 2023).

#### 2.4.4 Registration

For each participant, these GM masks were aligned from standard MNI space to native diffusion space using the following registration steps. We first used FSL's *epi_reg* tool to align the DWI with no diffusion‐weighting applied (i.e., the mean b0 image) to the high‐resolution T1‐weighted anatomical image volume using a boundary‐based registration with six degrees of freedom (Greve and Fischl 2009). We then inverted the resulting transformation matrix and applied this transformation matrix to align the high‐resolution T1‐weighted anatomical image volume to the mean b0 image using FSL's *flirt* with 12 degrees of freedom. Next, we aligned the high‐resolution T1‐weighted anatomical image volume, in DWI space, to the standard, skull‐stripped FSL MNI 152 1 mm^3^ template using FSL's *flirt* with 12 degrees of freedom. We inverted the resulting transformation matrix and applied this transformation matrix to align each of the eight network masks to the mean b0 image using FSL's *flirt* with 12 degrees of freedom and the *nearestneighbour* interpolation. A trained researcher visually inspected the quality of alignments and mask coverage for all ROIs and confirmed that all masks were of usable quality.

#### 2.4.5 Extracting Diffusion Metrics

Before extracting NODDI diffusion metrics of GM microstructure, a whole‐brain GM mask was generated using the *5ttgen* tool in MRtrix, thresholded to only include voxels that were classified as GM with > 50% probability, and then binarized. Each ROI mask was multiplied by this binary whole‐brain GM mask to exclude voxels containing WM or CSF. All ROI masks were further limited to voxels with intracellular diffusion < 0.99 to account for artifactual, mathematical errors in regions with insufficient signal (Emmenegger et al. 2021). Because NODDI can overestimate the fraction of free water diffusion, which in turn biases the estimation of the tissue parameters (Alsameen et al. 2023), especially in WM tissue (Gong et al. 2020), the intracellular and dispersed diffusion tissue compartment outputs were further thresholded to exclude voxels with free diffusion > 90% to avoid unreliable voxels with low tissue content, as in our prior work (Greenman and Bennett 2025; Venkatesh et al. 2021). Finally, for each participant, the resulting masks were then separately multiplied by each NODDI diffusion map (intracellular, dispersed, and free) and values were averaged across voxels within each mask.

### 2.5 Statistical Analyses

Statistical analyses were conducted using R Studio (v 4.3.1). To identify the underlying structure of the 12 measures of cognitive performance described previously (in Section 2.2), we conducted an exploratory factor analysis (EFA). Because the 12 individual variables comprised different scales of measurement, all variables were standardized before further analysis. First, we checked whether the data were appropriate for factor analysis using the Kaiser–Meyer–Olkin (KMO) method; we evaluated the measure of sample adequacy (MSA) for the overall dataset and each individual variable (using the *KMO* function in the *psych* package). We used MSA values > 0.80 as evidence that the data were well‐suited for running a factor analysis (Lorenzo‐Seva and Ferrando 2021). We used Barlett's test for sphericity (*cortest.bartlett* function), to compare the data correlation matrix to an identity matrix (i.e., a matrix filled with zeroes). Parallel analysis of eigenvalues was used to determine how many factors to extract (*fa.parallel* function). Finally, based on the number of factors suggested by the parallel analysis, we conducted an EFA using an orthogonal varimax rotation (*fa* function in the *psych* package), which helps make factors clearer by reducing cross loadings and minimizing smaller loading values. Individual tests that did not load sufficiently onto a single factor (with a loading of at least 0.60) were excluded from further interpretation. Age‐related differences in the resulting factor scores were then tested used linear regressions, with years of education completed included as a covariate, and were corrected for multiple comparisons using false discovery rate (FDR) procedures (Benjamini and Hochberg 1995).

A Head Coil (8‐channel or 48‐channel) × Age Group (younger [18–39 years of age], middle‐aged [40–59 years of age], and older [60–88 years of age]) *χ* ^2^ test indicated that the distribution of age group did not significantly differ across the two scanner configurations, *χ* ^2^ = 2.412, *p* = 0.299. Nonetheless, to help account for potential effects of the scanner upgrades (described under Section 2.3), we used version 2.4.5 of the *neuroHarmonize* Python tool to harmonize the NODDI GM microstructure data (Pomponio et al. 2020). The two scanner configurations were treated as the site variable, and age and sex were included as covariates. Age‐related differences in the harmonized estimates of GM microstructure were assessed using linear regressions, separately for each of the eight networks and each NODDI measure (the intracellular, dispersed, or free diffusion metric). Results were corrected for multiple comparisons using FDR procedures.

For both the cognitive and GM microstructure data, we tested whether age‐related differences in these measures were better explained by a nonlinear than linear model. We conducted additional linear regressions between chronological age + age‐squared and each outcome measure of interest. We then compared model fit between linear (age) and nonlinear (age + age‐squared) models, with better fit indicated by significant likelihood ratio tests (using the *lrtest* function).

Finally, we used Model 4 of the PROCESS macro (Hayes and Rockwood 2017) to test whether GM microstructure statistically mediated the relation between age and the cognitive factor scores. These analyses were conducted separately for each network, but with each NODDI diffusion measure from that network that was significantly related to age included as a parallel mediator. We considered the statistical mediation effects to be significant if their 95% confidence intervals (CIs) did not contain zero after 10,000 bootstrap replacements. We also used Model 1 of the PROCESS macro to test whether age moderated the relation between GM microstructure and the cognitive factor scores. Moderation effects were considered significant if there was an uncorrected *p*‐value < 0.05 for the interaction term, and we followed up on these interactions using the Johnson–Neyman technique (Bauer and Curran 2005; Johnson and Fay 1950). Years of education completed was included as a covariate for these analyses including cognition.

## 3 Results

### 3.1 Age‐Related Differences in Cognitive Performance

Adequacy of the data for factor analysis was confirmed using the KMO method, which indicated an overall MSA value = 0.890. No individual variable exhibited an MSA value < 0.800, together indicating that the data were well‐suited for factor analysis. The result from Barlett's test for sphericity was also significant, *χ* ^2^(66) = 846.55, *p* < 0.001, indicating that the variables were sufficiently correlated for factor analysis. A parallel analysis determined that a two‐factor solution was appropriate for the data. We then conducted a two‐factor EFA, using an orthogonal varimax rotation. This two‐factor model yielded an accepted fit to the data, *χ* ^2^(43) = 56.71, *p* = 0.08, root mean square error of approximation (RMSEA) = 0.045, with a Tucker–Lewis index of factoring reliability = 0.973.

Loadings and reliabilities of the variables on the factors and correlations between the factors, as well as the association between each variable and age, are presented in Table 2. Results indicated one factor primarily reflecting speed‐dependent, fluid cognition and a second factor reflecting episodic memory. The Flanker score, trail making test, and nonverbal working memory variables did not load sufficiently onto either factor and were excluded from further interpretation (loadings < 0.461).

**TABLE 2:** Results of the exploratory factor analysis (EFA).

Variable	Factor		Raw *α*	Age *r* ***
	Fluid cognition	Episodic memory		
Digit symbol substitution	0.646		0.876	−0.67
NIH card sort task	0.676		0.878	−0.49
Neutral Flanker RT	0.848		0.857	−0.72
Simple choice RT	0.620		0.898	−0.29
Choice RT	0.869		0.857	−0.66
NIH pattern comparison	0.672		0.880	−0.53
CVLT delayed recall		0.752	0.612	−0.43
WAIS logical memory		0.621	0.759	−0.27
NIH picture sequence		0.712	0.636	−0.62
Flanker score	—	—	—	−0.34
Trail making test	—	—	—	−0.40
Nonverbal working memory	—	—	—	−0.49

*Note:* Reliability estimates were based on Cronbach's raw *α* coefficient. The Flanker score, trail making test, and nonverbal working memory variables did not load sufficiently onto either factor, and their loadings and reliabilities are therefore excluded from further interpretation.

Abbreviations: CVLT = California Verbal Learning Test, NIH = National Institutes of Health, RT = reaction time, WAIS = WAIS–III Wechsler Adult Intelligence Scale, Third Edition.

*** *p* < 0.001.

**FIGURE 1:**
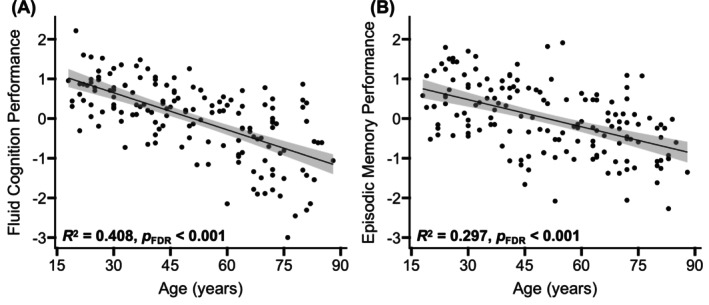
Age‐related differences in cognitive performance. A two‐factor solution derived from an EFA of 12 individual cognitive tests indicated that one factor primarily reflected tests of general fluid, speed‐dependent cognition (A) and a second factor reflected tests of episodic memory (B). Linear regressions indicated that significant age‐related differences were evident in both factors after applying FDR correction. The shaded gray areas around the regression lines represent 95% confidence intervals.

We assessed linear and nonlinear age‐related differences in cognitive performance using linear regressions with age or age and age‐squared predicting each cognitive factor score (FDR‐corrected for two comparisons), with years of education completed included as a covariate. Results indicated that older adult age was a significant predictor of lower performance for both domains, adjusted *R* ^2^ ≥ 0.297, *p*_FDR_ ≤ 0.001 (Figure 1).

We then used likelihood ratio tests to compare the degree of fit of the linear and nonlinear models for each factor. Results indicated that a nonlinear model did not account for significantly for age‐related variance than a linear model, for either factor, *χ* ^2^ ≤ 0.73, *p*_FDR_ ≥ 0.79.

### 3.2 Age‐Related Differences in GM Microstructure

Next, we assessed linear and nonlinear age‐related differences in GM microstructure using linear regressions, with either age alone (linear) or age and age‐squared (nonlinear) predicting each NODDI measure (the intracellular, dispersed, or free diffusion metric), with separate regression models for the dorsal attention, default mode, frontoparietal, limbic, sensorimotor, subcortical, ventral attention, or visual network (FDR‐corrected for eight comparisons). Likelihood ratio tests were used to compare the degree of fit of the linear and nonlinear models per network.

#### 3.2.1 Intracellular Diffusion

**FIGURE 2:**
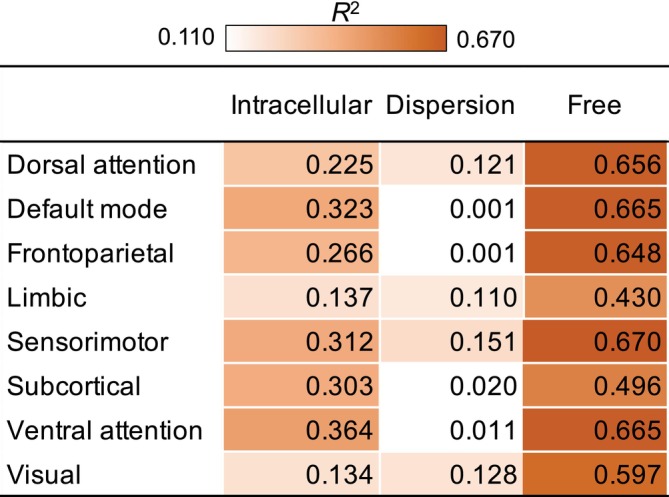
Summary of age‐related differences in GM microstructure. For each NODDI measure of GM microstructure and each network of interest, the coefficients of determination (*R* ^2^) are color‐coded using a heatmap to illustrate the magnitude of age‐related differences. The coefficients of determination are reported from the model that was a better fit for that network and diffusion metric (linear or nonlinear). Lighter‐orange cells correspond to weaker effects, darker‐orange cells correspond to stronger effects, and white/uncolored cells correspond to nonsignificant effects.

**FIGURE 3:**
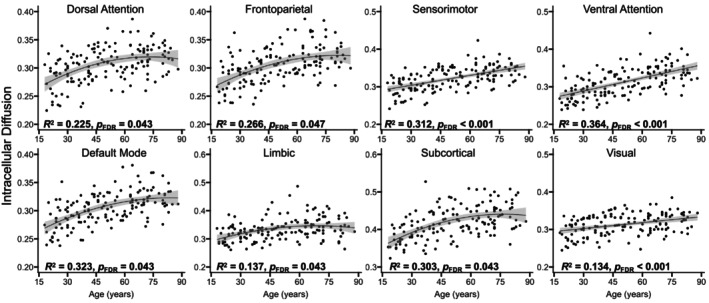
Age‐related differences in intracellular diffusion. Scatterplots display the relations between age and GM microstructure, as assessed by intracellular diffusion, across the adult lifespan, separately for each network. The regression line and coefficients of determination (*R* ^2^) reflect whether the linear (straight line) or nonlinear (curved line) analysis were a better fit, as determined by significant likelihood ratio tests. The shaded gray areas around the regression lines represent 95% confidence intervals, and significant effects are presented in bold. FDR = false discovery rate.

Results indicated that older adult age was a significant predictor of higher intracellular diffusion for all eight networks, adjusted *R* ^2^ ≥ 0.110, *p*_FDR_ ≤ 0.001 (Figures 2 and 3). A nonlinear model was a significantly better fit for explaining age‐related increases in intracellular diffusion for the dorsal attention, default mode, frontoparietal, limbic, and subcortical networks, *χ* ^2^ ≥ 4.02, *p* ≤ 0.04 (Figure 3).

#### 3.2.2 Dispersed Diffusion

**FIGURE 4:**
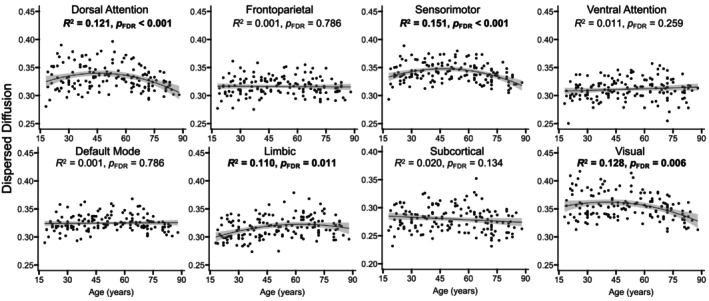
Age‐related differences in dispersed diffusion. Scatterplots display the relations between age and GM microstructure, as assessed by dispersed diffusion, across the adult lifespan, separately for each network. The regression line and coefficients of determination (*R* ^2^) reflect whether the linear (straight line) or nonlinear (curved line) analysis were a better fit, as determined by significant likelihood ratio tests. The shaded gray areas around the regression lines represent 95% confidence intervals, and significant effects are presented in bold. FDR = false discovery rate.

Results indicated that older adult age was a significant predictor of lower dispersed diffusion in the dorsal attention, sensorimotor, and visual networks, and higher dispersed diffusion in the limbic network, adjusted *R* ^2^ ≥ 0.033, *p*_FDR_ ≤ 0.05 (Figures 2 and 4), and a nonlinear model was a significantly better fit for explaining age‐related differences in dispersed diffusion for each of these four networks, χ^2^ ≥ 6.68, *p* ≤ 0.01 (Figure 4). There were no significant age‐related differences in dispersed diffusion for the default mode, frontoparietal, subcortical, or ventral attention networks, adjusted *R* ^2^ ≤ 0.020, *p*_FDR_ ≥ 0.13 (Figure 4).

#### 3.2.3 Free Diffusion

**FIGURE 5:**
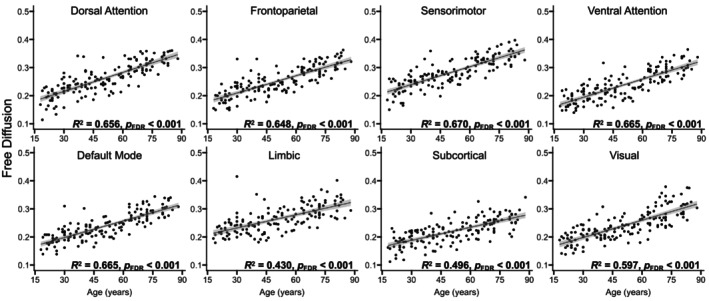
Age‐related differences in free diffusion. Scatterplots display the relations between age and GM microstructure, as assessed by free diffusion, across the adult lifespan, separately for each network. The regression line and coefficients of determination (*R* ^2^) reflect that a linear model was a better fit than the nonlinear model for all networks, as determined by significant likelihood ratio tests. The shaded gray areas around the regression lines represent 95% confidence intervals, and significant effects are presented in bold. FDR = false discovery rate.

Results indicated that older adult age was a significant predictor of higher free diffusion for all eight networks, *R* ^2^ ≥ 0.430, *p*_FDR_ ≤ 0.001 (Figures 2 and 5). A nonlinear model was not a significantly better fit than the linear model for explaining age‐related differences in free diffusion in any network, χ^2^ ≤ 3.05, *p* ≥ 0.08 (Figure 5).

### 3.3 GM Microstructure and Fluid Cognition

To test whether GM microstructure helped explain age‐related differences in cognitive performance, we conducted separate statistical mediation analyses for each network. The microstructure measures that were significantly related to age (Table 2) were included as parallel mediators of the relation between age and each cognitive factor score (general fluid cognition or episodic memory). Years of education completed was included as a covariate. Results indicated that the relation between age and episodic memory performance was partially statistically mediated by dispersed diffusion in the dorsal attention network (Table 3), accounting for 5.982% of the variance in this relation.

**TABLE 3:** Statistical mediation of episodic memory by dorsal attention microstructure.

	Effect	SE	*t*	*p*	Lower CI	Upper CI
Age effect (*a* path)						
Dispersed diffusion	**−0.0002**	**0.0001**	**−2.30**	**0.023**	**−0.0004**	**−0.0000**
Intracellular diffusion	**0.0007**	**0.0001**	**5.99**	**< 0.001**	**0.0005**	**0.0009**
Free diffusion	**0.0023**	**0.0001**	**16.90**	**< 0.001**	**0.0020**	**0.0025**
Mediator to outcome (*b* path)						
Dispersed diffusion	**−6.479**	**3.055**	**−2.12**	0.**036**	**−12.516**	**−0.441**
Intracellular diffusion	0.258	2.263	0.11	0.909	−4.214	4.730
Free diffusion	−0.849	1.981	−0.42	0.669	−4.764	3.067
Total effect for age (*c* path)						
	**−0.023**	**0.003**	**−7.64**	**< 0.001**	**−0.029**	**−0.017**
Direct effect for age (*c*′ path)						
	**−0.023**	**0.005**	**−4.24**	**< 0.001**	**−0.034**	**−0.012**
Mediation effect (*a* × *b* path interaction)						
Total	−0.004	0.004	—	—	−0.009	0.008
Dispersed diffusion	**0.001**	**0.001**	—	—	**0.000**	**0.003**
Intracellular diffusion	0.000	0.002	—	—	−0.003	0.007
Free diffusion	−0.002	0.004	—	—	−0.011	0.004

*Note:* Parallel mediation models with age as the predictor variable (*x*), the memory factor score as the outcome variable (*y*), and dispersed, intracellular, and free diffusion within the dorsal attention network as parallel mediators (*m*); *a* = predictor to mediator pathway; *b* = mediator to outcome pathway, while controlling for *a* path; *c* = total effect of predictor; *c′* = direct effect of predictor, controlling for mediators; *a × b* = interaction of *a* and *b* paths representing indirect influence of *x* on *y* as mediated by *m*; effect = unstandardized beta coefficient; lower/upper CI = lower/upper bounds of bias‐corrected 95% confidence intervals. Years of education completed was included as a covariate. Significant effects are presented in bold.

Finally, we tested whether the relation between GM microstructure and performance on each factor score was significantly moderated by age. Interaction terms with corresponding uncorrected *p*‐values < 0.050 were probed using the Johnson–Neyman technique. Analyses were again limited only to the measures of GM microstructure that were significantly related to age.

**FIGURE 6:**
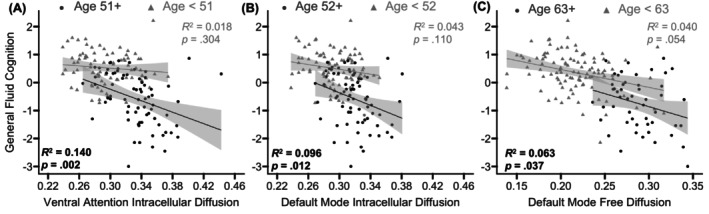
Microstructure‐cognition relations. For the networks in which the relation between a GM microstructure measure and the general fluid cognition factor score were moderated by age, the corresponding GM microstructure measure is plotted against cognitive performance, separately for participants above or below the Johnson–Neyman inflection point. Lower general fluid cognition performance was related to higher intracellular diffusion within the ventral attention network for adults beyond 51 years of age (A) and the default mode network for adults beyond 52 years of age (B), and to higher free diffusion in the default mode network for adults beyond 63 years of age (C). The shaded gray area around the regression lines represents 95% confidence intervals. Significant effects are presented in bold.

When fluid cognition was the outcome variable, significant interaction terms were observed for intracellular diffusion within the ventral attention, *t*(148) = −2.49, *p* = 0.014, and default mode, *t*(148) = −2.02, *p* = 0.045, networks, and for free diffusion within the limbic, *t*(148) = −2.23, *p* = 0.028, and default mode, *t*(148) = −2.01, *p* = 0.046, networks. Follow‐up Johnson–Neyman analyses indicated that worse general fluid cognition performance was related to higher intracellular diffusion within the ventral attention (Figure 6A) and default mode (Figure 6B) networks but only for adults beyond 51 and 52 years of age, respectively, and to higher free diffusion in the default mode network for adults beyond 63 years of age (Figure 6C). The relation between fluid cognition and free diffusion in the limbic network was not significant for any age range and was driven by a crossover effect where the relation was positive for younger adults but negative for older adults.

## 4 Discussion

Previous investigations of GM microstructure in healthy aging have mostly been limited to extreme age group designs or only samples of older adults and did not comprehensively examine all NODDI metrics or multiple aspects of cognition. We conducted multicompartment modeling of DWI data to assess age‐related differences in GM microstructure across the adult lifespan and to determine whether GM microstructural alterations accounted for age‐related variability in measures of fluid cognition and episodic memory. We observed that the intracellular and dispersed diffusion metrics primarily exhibited nonlinear age‐related increases and decreases, respectively, whereas the free diffusion metric exhibited strikingly linear age‐related increases. The age‐related differences in GM microstructure metrics were behaviorally relevant, such that they helped explain differences in episodic memory across the lifespan (dispersed diffusion) and differences in fluid cognition among middle‐age and older adults (intracellular and free diffusion). These findings extend the cortical disconnection hypothesis by suggesting that structural alterations within individual GM regions contribute to age‐related cognitive decline across the adult lifespan, comparable to what has been observed for the disconnection of WM pathways between these regions (e.g., Agah et al. 2025; Madden et al. 2009, 2017; Pang et al. 2025; Peter et al. 2025).

When examining effects of age on each diffusion metric, we observed the strongest and linear age‐related increases for free diffusion (Figure 5), followed by relatively weaker and predominantly nonlinear age‐related increases for intracellular diffusion (Figure 3) and decreases for dispersed diffusion (Figure 4). Our use of a biophysically informed model extends previous work examining GM microstructure using model‐free mathematical frameworks (Bouhrara et al. 2023; Singh et al. 2024). The direction of the observed age‐related patterns as well as the relative magnitude across NODDI metrics replicates our prior work in healthy younger and older adults (Greenman and Bennett 2025), with the current study providing novel information about potential neurobiological substrates given the linearity of GM microstructural aging. That is, our findings for the free diffusion metric suggest that it captures GM properties that exhibit a monotonic difference throughout the brain starting in early adulthood, such as linear declines observed for neuronal shrinkage or loss (Andersen et al. 1999) or gradual accumulation of Alzheimer's disease‐related neuropathologies (Nakaya et al. 2022; Schifani et al. 2025; Wang et al. 2022; Yu et al. 2024). In contrast, the intracellular diffusion metric captures GM properties that differ through late‐middle age and plateau in older adulthood, whereas GM properties captured by the dispersed diffusion metric peak in late‐middle age and decline in older adulthood. Cortical myelin development may contribute to the initial increase in the intracellular diffusion metric as it has been shown to increase through middle age (Bouhrara et al. 2021; Shafee et al. 2015), although it degrades (rather than plateaus) in older age (Grydeland et al. 2013; Khattar et al. 2021). Similarly, dendritic complexity may explain the initial increase in the dispersed diffusion metric as it decreases from early to middle age, but it then plateaus (rather than increases) in older adulthood (de Brabanr et al. 1998; Dickstein et al. 2013; Duan et al. 2003; Jacobs et al. 1997). We speculate that additional late‐onset neural mechanisms (e.g., cerebrovascular disease, inflammation) explain the effects observed for these NODDI metrics in older age. Confirming this speculation would be an important direction for future mechanistic research aimed at characterizing aging of GM microstructure across the adult lifespan, such as high‐gradient DWI studies that have reported age‐related differences in soma‐specific sources of diffusion within GM tissue (Lee et al. 2024; Singh et al. 2025).

Regarding the region‐specificity of the tissue‐compartment NODDI metrics, age‐related increases in the intracellular diffusion metric were nonlinear in association networks (i.e., dorsal attention, default mode, frontoparietal, limbic, ventral attention) and the subcortical network, but linear in sensory networks (i.e., sensorimotor, visual), whereas age‐related decreases for the dispersed diffusion metric were predominantly nonlinear and limited to only half of the networks (i.e., dorsal attention, limbic, sensorimotor, and visual networks). Finding the strongest age‐related effects for the intracellular diffusion metric in networks comprising frontoparietal regions is consistent with prior structural and functional MRI studies that report greater vulnerability of association than sensory networks in healthy aging (Chan et al. 2014; Wu et al. 2016; Zhang et al. 2023). Whereas one prior NODDI study that included adults across the lifespan found no significant age‐related differences in the intracellular diffusion metric (Nazeri et al. 2015), this discrepancy might reflect their relatively small number of participants per age decade (overall *n* = 45) or their use of the default intrinsic diffusivity parameter, which we previously demonstrated influences the magnitude and direction of age effects (Greenman and Bennett 2025). A strength of our current analyses is the use of an intrinsic diffusivity parameter optimized for GM rather than WM (Fukutomi et al. 2019, 2018; Guerrero et al. 2019) and the acquisition of a sample nearly three times larger, providing more statistical power to examine the linearity of age‐related effects on GM microstructure in adults across the lifespan.

By assessing both general fluid cognition and episodic memory in the same study, we were able to test whether distinct GM microstructural properties were uniquely related to each cognitive domain. As expected, significant linear age‐related decreases were evident in factor scores representing both domains (Figure 1), with a stronger age‐related effect observed for fluid, speed‐dependent cognition than episodic memory. We further observed that worse fluid cognition performance was related to increases in the intracellular diffusion metric within the default mode and ventral attention networks for adults beyond 51 years of age and to increases in the free diffusion metric within the default mode network for adults beyond 63 years of age (Figure 6). This study is the first to show that chronological age moderates the relation between GM microstructure and cognitive performance and may suggest that a sufficient degree of age‐related alterations must occur before GM microstructure affects cognitive ability. Prior work similarly observed that GM microstructure (dispersed diffusion) in the hippocampus and frontal pole was related to performance on a more limited outcome measure of executive function (Stroop test, Trail Making Test part *B*, and letter‐number sequencing; Nazeri et al. 2015). Together, these findings complement those from studies employing advanced WM imaging techniques, which have similarly observed that age‐related changes in myelin water fraction longitudinally explains declines in fluid cognitive abilities (Gong et al. 2024, 2023).

In contrast to the moderation effects observed for fluid cognition, we instead found that the dispersed diffusion metric within the dorsal attention network was a partial statistical mediator of age‐related differences in episodic memory (Table 3). The dorsal attention network comprises regions like the precuneus and the superior and inferior parietal lobules, which have been theorized to contribute to the attentional demands of episodic memory (Cabeza et al. 2008). This network also includes the dorsolateral prefrontal cortex, a region where intracellular diffusion has been related to episodic memory performance in healthy older adults and patients with mild cognitive impairment (Gozdas et al. 2021). Together with prior work, the current findings have implications for cortical disconnection theories of aging by suggesting that structural alterations within GM regions themselves contribute to age‐related cognitive decline, in addition to the disconnection of WM fiber bundles between these distributed regions (Bartzokis et al. 2004; Bennett and Madden 2014; O'Sullivan et al. 2001).

## 5 Limitations and Future Directions

One limitation of the current study is that we combined all subcortical regions into a single GM network mask, as in prior studies (Capogna et al. 2022; Luo et al. 2020; Merenstein, Zhao, et al. 2023). However, hippocampus and basal ganglia regions exhibit distinct microstructural alterations in healthy aging that cannot be disentangled here (Franco et al. 2021; Greenman and Bennett 2025; Ibrahim and Bennett 2023; Zia et al. 2025). Although the current findings help characterize which aspects of GM microstructure differ in healthy aging, they do not address why and how these microstructural alterations occur. Future work should try to better understand the factors moderating age‐related differences in GM microstructure, such as the role of sleep, cardiorespiratory fitness, and physical activity levels (Callow et al. 2023, 2025; Chylinski et al. 2022; Mueller et al. 2024). Because the current findings were derived from a sample of participants that largely identified as non‐Hispanic white, further studies are needed to determine how trajectories of GM aging may vary across adults from different demographic and socioeconomic backgrounds, especially those employing longitudinal designs.

Despite its increased biological specificity over traditional tensor‐based models, NODDI is not without limitations. For one, NODDI is based on a more standard biophysical model, where specific parameters values are fixed to prioritize precision and may not always accurately map onto the underlying neurobiology (Jelescu et al. 2016). In particular, NODDI assumes that neurite water diffusion in GM occurs along a collection of impermeable sticks (membranes), but this assumption is less valid for GM than WM tissue (Jelescu et al. 2022, 2016). Using relatively lower gradients, as in the current study (i.e., ≤ 3000 s/mm^2^), NODDI is generally successful at modeling water diffusion in different cellular compartments (Palombo et al. 2020). However, to more accurately capture the properties of GM tissue, other advanced approaches have added an additional impermeable, spherical soma compartment using high‐gradient data acquisitions (soma and neurite density imaging; Palombo et al. 2020) or explicitly modeled water exchange between compartments (Neurite Exchange Imaging; Jelescu et al. 2022).

Other concerns include the assumption of equivalence between intrinsic and extrinsic diffusivity parameter values (Jelescu et al. 2020) and the overestimation of the fraction of free water diffusion, which in turn biases the estimation of the tissue parameters (Alsameen et al. 2023). We tried to assuage these concerns here by modifying the intrinsic diffusivity parameter to a value better aligned for the intricate properties of GM tissue (Crombe et al. 2018; Fukutomi et al. 2019, 2018; Greenman and Bennett 2025; Guerrero et al. 2019) and excluding voxels with > 90% free water content from tissue‐specific measures (Greenman and Bennett 2025; Venkatesh et al. 2021). Despite having increasing biological specificity in vivo, NODDI (and traditional tensor‐based measures) continue to face challenges with differentiating intracortical layers in samples of ex vivo brain tissue (Kundu et al. 2023). Overall, the field will benefit from future studies combining in vivo analyses with ex vivo histological validation so that the optimal choice of model parameters for GM tissue can continue to be refined, especially for data acquired from clinical scanners.

## 6 Conclusions

In closing, we observed significant age‐related alterations in GM microstructure as large linear increases in free diffusion, moderate nonlinear increases in intracellular diffusion, and fewer significant age‐related effects for dispersed diffusion. These GM microstructural alterations in the default mode and ventral attention networks were related to worse fluid cognition, but only for middle‐age and older adults, whereas dispersed diffusion helped explain differences in memory performance across the adult lifespan. Together, these results have important theoretical implications by suggesting that healthy aging is accompanied by unique profiles of GM microstructural alterations that affect cognitive abilities, especially toward the latter half of the adult lifespan.

## Funding

This work was supported by the National Institute on Aging (R01 AG039684).

## Conflicts of Interest

The authors declare no conflicts of interest.

## Data Availability

The deidentified behavioral and preprocessed neuroimaging data from are available upon reasonable request to the corresponding author and a data sharing agreement will be required. The code that was used for data analyses will be made available on the GitHub profile of the corresponding author upon manuscript acceptance.
